# The Influence of Si/Al Ratios on Adsorption and Desorption Characterizations of Pd/Beta Served as Cold-Start Catalysts

**DOI:** 10.3390/ma12071045

**Published:** 2019-03-29

**Authors:** Ming Jiang, Jun Wang, Jianqiang Wang, Meiqing Shen

**Affiliations:** 1 Key Laboratory for Green Chemical Technology of State Education Ministry, School of Chemical Engineering & Technology, Tianjin University, Tianjin 300072, China; jiangming@tju.edu.cn (M.J.); wangjun@tju.edu.cn (J.W.); jianqiangwang@tju.edu.cn (J.W.); 2 Collaborative Innovation Center of Chemical Science and Engineering (Tianjin), Tianjin 300072, China; 3 State Key Laboratory of Engines, Tianjin University, Tianjin 300072, China

**Keywords:** cold start, Pd/Beta, Si/Al ratios, NO_x_ and C_3_H_6_ adsorption, low temperature desorption

## Abstract

The majority of NO_x_ is exhausted during the cold-start period for the low temperature of vehicle emissions, which can be solved by using Pd/zeolite catalysts to trap NO_x_ at low temperature and release NO_x_ at a high temperature that must be higher than the operating temperature of selective catalytic reduction catalysts (SCR). In this work, several Pd/Beta catalysts were prepared to identify the influence of Si/Al ratios on NO and C_3_H_6_ adsorption and desorption characterizations. The physicochemical properties were identified using N_2_ physical adsorption, Fourier Transform Infrared Spectroscopy (FT-IR), transmission electron microscopy (TEM), X-ray photo electron spectroscopy (XPS), and Na^+^ titration, while the adsorption and desorption characterizations were investigated by catalyst evaluation. The results indicated that the amount of dispersed Pd ions, the main active sites for NO and C_3_H_6_ adsorption, decreased with the increase of Si/Al ratios. Besides this, the intensity of Brønsted and Lewis acid decreased with the increase of Si/Al ratios, which also led to the decrease of NO and C_3_H_6_ adsorption amounts. Therefore, Pd dispersion and the acidic properties of Pd/Beta together determined the adsorption ability of NO and C_3_H_6_. Moreover, lower Si/Al ratios resulted in the formation of an additional dispersed Pd cationic species, Pd(OH)^+^, from which adsorbed NO released at a much lower temperature. Finally, an optimum Si/Al ratio of Pd/Beta was found at around 55 due to the balanced performance between the adsorption amounts and desorption temperature.

## 1. Introduction

Regulations on NO_x_ emission of vehicles are getting much more stringent [1,2,3,4]. So, it is a significant challenge to reduce NO_x_ emission and satisfy further regulations for the automotive industry. It is reported that the majority of NO_x_ is exhausted during the cold-start period of vehicles. In this period, the temperature of vehicle emissions is much lower than operating-temperature ranges of SCR (urea-based selective catalytic reduction catalysts) and NSR (NO_x_ storage and reduction catalysts) [5,6], which results in low efficiency of these two NO_x_ emission control technologies in this period.

In order to solve the problem of NO_x_ emission in the cold-start period, Chen et al. [7] proposed using Pd/zeolite served as passive NO_x_ absorbers (PNAs) for the first time. This catalyst exhibits a high capacity of NO_x_ adsorption and suitable desorption temperature for downstream NH_3_-SCR catalysts. Besides this, Pd/zeolite can also adsorb hydrocarbons during the cold-start period. So Pd/zeolite is regarded as a novel cold-start catalyst in the next generation. Further studies were done to investigate Pd species and active sites for NO trapping by Gao et al. [8]. They suggested that atomically dispersed Pd ions and aggregated palladium oxide coexisted in this catalyst. Highly dispersed Pd ions at exchange sites of zeolites were believed to be the active site for the low temperature NO storage [7,8,9,10]. 

Since the dispersion of Pd loaded on Pd/zeolite is significantly determined by Si/Al ratios [11,12,13], it is necessary to investigate the influence of the Si/Al ratio on adsorption and desorption characterizations of Pd/zeolite. Lee et al. [14] suggested that the high Al concentration on Pd/ZSM-5 provided a high NO adsorption ability and also suppressed the PdO sintering. Mihai et al. [15] investigated the effect of the Si/Al ratio on NOx adsorption performance in the absence and presence of CO for Pd/BEA and Pd/SSZ-13, and they found the presence of CO was only beneficial for Pd/zeolites with low and medium Si/Al ratios. However, in view of the presence of hydrocarbons in the automotive exhaust, the addition of C_3_H_6_ in the adsorbed gases, which can significantly change adsorption and desorption characterizations, is necessary to select a suitable Si/Al ratio of Pd/zeolite for a cold-start application.

In this study, Pd species of Pd/Beta catalysts with different Si/Al ratios were characterized using Fourier Transform Infrared Spectroscopy (FT-IR), transmission electron microscopy (TEM), and X-ray photo electron spectroscopy (XPS), and different Pd species were quantified through Na^+^ titration. This study aims to gain insights into the influence of Si/Al ratios on the dispersity of Pd species and on the adsorption and desorption characterization of Pd/Beta. The reason for earlier NO desorption in low Si/Al ratio samples was explained and high-temperature reactions for adsorbed gases in the desorption stage were also studied. Finally, a suitable Si/Al ratio of Pd/zeolite for cold-start application was obtained.

## 2. Experimentation

### 2.1. Catalyst Preparation

H-Beta zeolites with Si/Al ratios of 16, 55, and 83 were provided by Novel Chemistry (Shanghai, China). Samples with palladium loading were prepared using incipient wetness impregnation with Pd(NO_3_)_2_ solution (15.47 wt.% Pd, Heraeus Materials Technology Shanghai Ltd., Shanghai, China). Then, these samples were dried at 100 °C overnight and further calcined at 550 °C for 4 h in air. All samples were stabilized via the 12-h hydrothermal treatment at 750 °C in air with 10% H_2_O. Pd loading was measured by inductively coupled plasma (ICP) analysis (Agilent 5100 ICP-OES, Agilent Technologies, Santa Clara, CA, USA). The Si/Al ratio was detected by X-ray fluorescence (XRF, HD-S4 Pioneer, Bruker, Billerica, MA, USA) analysis. These samples were named as Pd/x where x represented Si/Al ratios of the corresponding parent H-Beta. Pd loading and Si/Al ratios of each sample were exhibited in Table 1.

### 2.2. Catalyst Characterization

N_2_ physical adsorption was performed with a sorption analyzer (ASAP 2460 Micromeritics Instrument Co., Norcross, GA, USA) to characterize the porous texture of each sample. All samples were previously degassed at 300 °C for 10 h. The specific surface areas (S_BET_) of all samples were calculated by the Brunner-Emmet-Teller (BET) method and the micropore volume (V_micro_) was calculated by the t-Plot method.

To observe the morphology features of each sample, high resolution transmission electron micrograph (HRTEM) figures were obtained through JEM-2100F Field Emission Electron Microscope (JEOL, Akishima, Tokyo).

To confirm the existence of Pd ions at exchange sites of zeolites, ex-situ FT-IR spectra were obtained on a Nicolet iS10 FT-IR spectrometer (Thermo Fisher Scientific, Waltham, MA, USA). KBr was used to collect the background spectrum. All samples were preoxidized in 10% O_2_ balanced with N_2_ at 500 °C for 30 min. Spectra were collected at 200 °C in N_2_.

Na^+^ titration was used to measure the amount of isolated Pd ions. The powder was stirred with 0.1M NaCl (purity above 99.5%, Shanghai Zhenpin Chemical Co., Ltd., Shanghai, China) solution for 4 h. Then, samples were washed via suction filtration. After that, the powders obtained were dried at 100 °C for 6 h. The above operations were repeated three times.

Pd species were further probed by CO through in situ FT-IR on a Nicolet iS10 FT-IR spectrometer. Samples were preoxidized in 10% O_2_ balanced with N_2_ at 500 °C for 30 min. CO served as the probe molecule was stabilized in the bypass and then introduced into the sample cell at 80 °C. Spectra were collected at 80 °C in 1000 ppm CO balanced with N_2_.

X-ray photo electron spectroscopy (XPS) was performed using Thermo ESCALAB 250XI (Thermo Fisher Scientific, Waltham, MA, USA) to characterize the chemical state of Pd. The excitation source was Al Kα radiation operated at 15 KV and 10 mA.

The existence of Lewis and Brønsted acid sites was probed by in situ FT-IR of NH_3_ adsorption on a Nicolet iS10 FT-IR spectrometer. Samples were preoxidized in 10% O_2_ balanced with N_2_ at 500 °C for 30 min. NH_3_ served as the probe molecule; it was stabilized in the bypass and then introduced into the sample cell at 80 °C. Spectra were collected at 80 °C in 500 ppm NH_3_ balanced with N_2_.

### 2.3. Catalyst Evaluation

In the catalyst evaluation, a 0.25 g sample (60–80 mesh) mixed with 0.75 g quartz (60–80 mesh) was put into a quartz reactor. The flow rate was 1 L/min. The sample was pretreated in the flow with 10% O_2_ balanced with N_2_ at 500 °C for 30 min and then was cooled to 80 °C in N_2_. In the meantime, the flow (200 ppm NO_x_, 167 ppm C_3_H_6_, 200 ppm CO, 5% CO_2_, 5% H_2_O, 10% O_2_, balanced with N_2_) was stabilized in the bypass and was introduced into the reactor when the temperature was stabilized at 80 °C. The adsorption stage continued for 180 s. After that, the desorption stage in N_2_ was observed with the increase of temperatures up to 500 °C. The ramping rate was 10 °C/min. Concentrations of each gaseous component were measured by an online MKS MultiGas 2030 FTIR gas analyzer (MKS Instruments, Andover, MA, USA) during the whole process. Besides this, a space velocity of 28,800 h^−1^ was adopted.

Multiple adsorption-desorption cycling experiments were performed to evaluate the regenerability of Pd-Beta catalysts. Catalyst evaluation processes were the same as described above. After the first cycle, the temperature decreased to 80 °C and then the next adsorption-desorption cycle was performed. The cycling experiments were performed six times.

## 3. Result

### 3.1. Catalyst Evaluation

**Figure 1:**
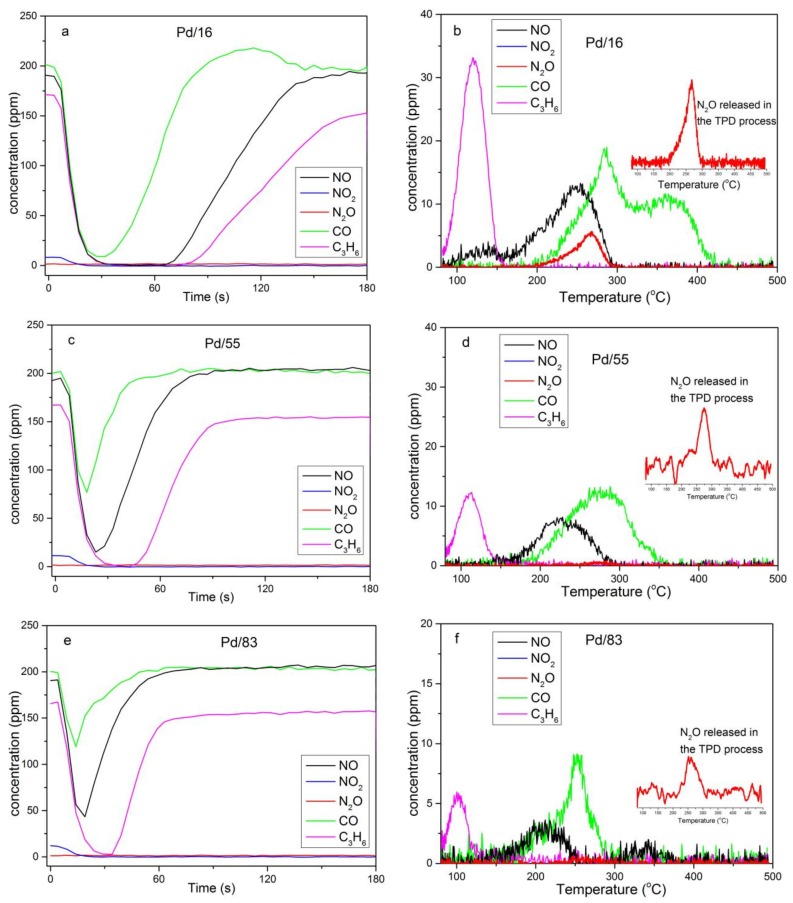
Catalyst evaluation of each sample (**a**, **c**, **e**: the adsorption stage in O_2_, CO_2_, N_2_, H_2_O, C_3_H_6_, CO, and NO_x_ (190 ppm NO, 10 ppm NO_2_) at 80 °C; **b**, **d**, **f**: the desorption stage in N_2_ up to 500 °C).

Catalyst evaluation results are exhibited in Figure 1. Adsorption amounts of each gaseous component were calculated through the integration of negative peaks in the adsorption stage at 80 °C (Table 2). The desorption amounts in the subsequent TPD experiment up to 500 °C (the desorption stage) were calculated through the same method (Table 2). As shown in Table 2, the adsorption amounts of NO and C_3_H_6_ decrease in parallel with the increase of Si/Al ratios. Considering the same Pd loading of each sample, different adsorption amounts of them indicate that not all Pd loaded on samples is activated. 

In the TPD experiment, C_3_H_6_ is desorbed at approximately110 °C, and the desorption of NO and CO is observed at 210–250 °C. Meanwhile, the formation of N_2_O is also observed in this temperature range. There is an extra NO desorption peak centered at 130 °C that cannot reach the operation temperature for downstream NH_3_-SCR catalysts, which leads these NO failures to be solved. Besides, the concentration of NO_2_ drops close to 0 ppm as soon as the adsorption stage begins, whereas no NO_2_ is released in the whole desorption stage on any sample. This phenomenon indicates NO_2_ reacts with other gases on Pd/Beta. 

As shown in Table 2, desorption amounts of NO and C_3_H_6_ decrease sharply and desorption amounts of CO increase compared with the adsorption amounts for all samples. Besides this, we can also see the formation of N_2_O in the desorption curves. Considering that Pd/zeolite can catalyze the reaction of NO and hydrocarbon (HC) [16,17,18,19], there might be NO reduction and C_3_H_6_ oxidation reaction in the desorption process. 

### 3.2. Adsorption–Desorption Cycling

**Figure 2:**
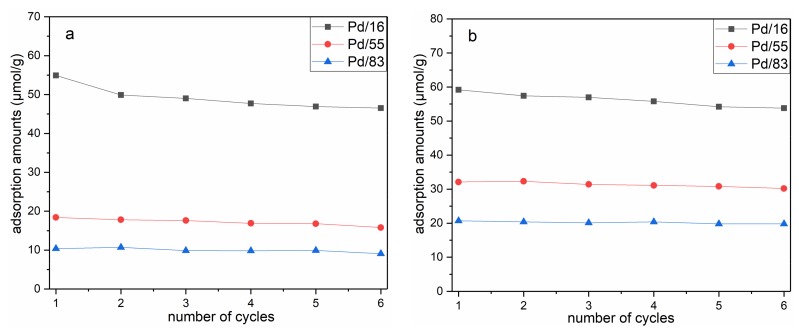
NO (**a**) and C_3_H_6_ (**b**) adsorption amounts for the adsorption–desorption cycles of each sample.

The durability of catalysts is crucial for real application [20,21], and the adsorbed gases must be released entirely before the next cycle for Pd/Beta catalysts. Multiple recycling experiments were performed to evaluate the regenerability of Pd/Beta catalysts and the results are exhibited in Figure 2. The NO adsorption performance of Pd/16 decreases significantly after the first cycle, while it decreases slightly in subsequent cycles. Besides this, the adsorption performance of Pd/55 and Pd/83 samples changes slightly in the cycling process. Overall, all samples can be regenerated by increasing the temperature to 500 °C.

### 3.3. Catalyst Characterization

#### 3.3.1. Textural Properties

Table 3 shows the textural properties of each sample. Specific surface areas and micropore volumes of the Pd/Beta and H-Beta samples slightly decrease with the increase of Si/Al ratios. Besides this, specific surface areas and the micropore volume of Pd/Beta samples are also slightly lower than those of the corresponding parent H-Beta, which is attributed to the partial collapse of channels caused by hydrothermal treatment. Figures S1 and S2 show N_2_ adsorption–desorption isotherms and pore size distribution of H-Beta and Pd/Beta. All samples present type Ⅰ isotherms and similar pore size distribution. 

#### 3.3.2. HRTEM

**Figure 3:**
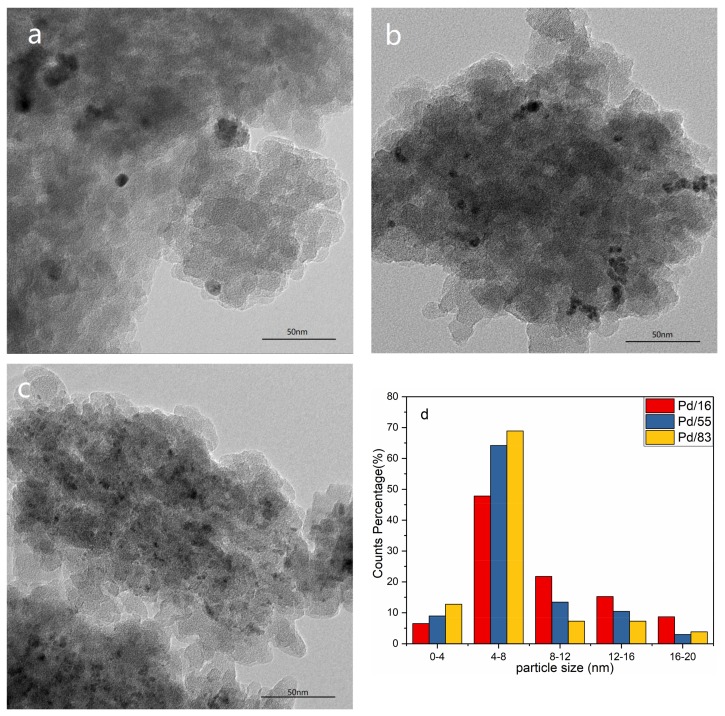
HRTEM figures of each sample ((**a**): Pd/16; (**b**): Pd/55; (**c**): Pd/83) and size distribution of PdO (**d**).

Considering the impregnation method adopted in the sample preparation, palladium oxide (marked as PdO) is likely to exist on the external surface of each sample. To observe the microstructure of PdO in each sample, HRTEM was performed and the results were displayed in Figure 3 and Figure S3. Inter-reticular distances of 2.153 Å, 2.644 Å, and 2.667 Å were measured in Figure S3. They are assigned to [110], [101], and [002] lattice fringes of PdO, respectively [22]. As shown in Figure 3, the PdO particle can be observed in each sample, and the amount of PdO particles increases with the increase of the Si/Al ratio, which means there are more dispersed Pd^2+^ at exchanged sites for the low Si/Al ratio sample. As shown in Figure 3d, all samples with different Si/Al ratios have similar PdO particle size distribution. 

#### 3.3.3. Ex-Situ FT-IR

**Figure 4:**
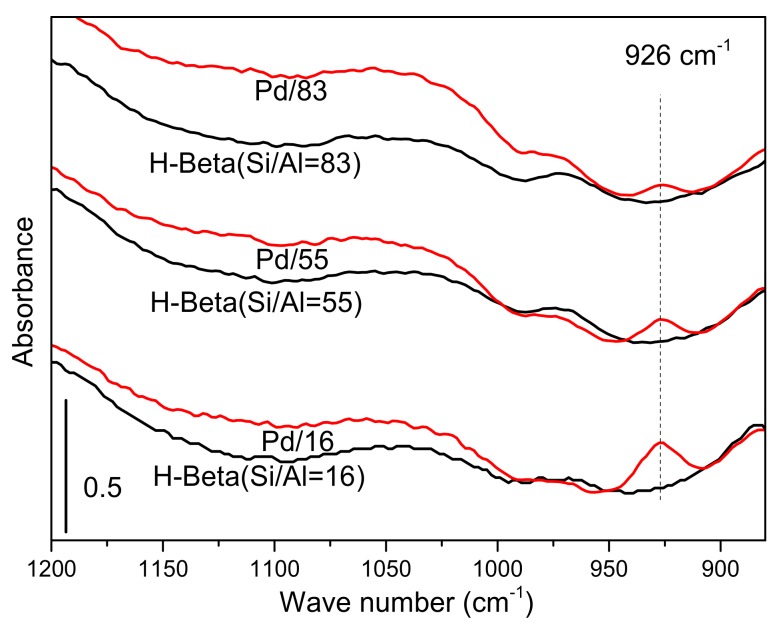
Ex-situ FT-IR of each sample (flow: N_2_; temperature: 200 °C).

To confirm the existence of Pd ions at exchange sites of zeolites, FT-IR spectra of Pd/16, Pd/55, Pd/83, and H-Beta with different Si/Al ratios were obtained, and the results are shown in Figure 4. Extra peaks at 926 cm^−1^ are observed on spectra of each Pd/Beta sample versus the corresponding parent H-Beta. It is reported that the vibration region from 800 cm^−1^ to 1000 cm^−1^ should be attributed to the distortion of the skeletal T–O–T bond caused by the interaction of cationic ions at exchange sites of zeolites [7,23]. So, the extra peak at 926 cm^−1^ clearly indicates the existence of Pd ions at exchange sites.

#### 3.3.4. In Situ FT-IR of CO Adsorption

**Figure 5:**
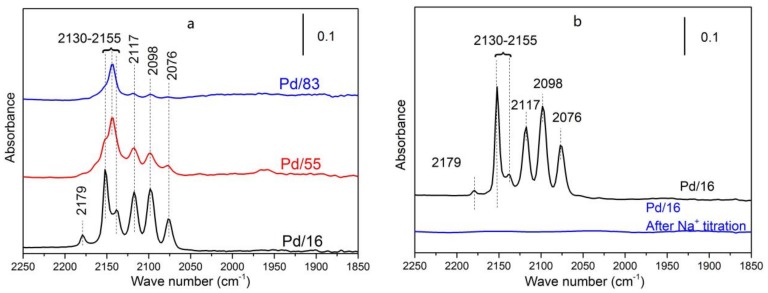
In situ FT-IR of CO adsorption (temperature: 80 °C; flow: CO (1000 ppm) + N_2_; (**a**): spectra of Pd/16, Pd/55, and Pd/83; (**b**): the spectrum of Pd/16 treated by Na^+^ titration).

Pd species are further probed by CO through in situ FT-IR. As shown in Figure 5a, peaks at 2098 and 2076 cm^−1^ are attributed to the C–O vibration on dispersed Pd^0^ [24] (marked as CO-Pd^0^). It is reported that the vibration region higher than 2100 cm^−1^ should be attributed to CO adsorbed on dispersed Pd ions at exchange sites of zeolites [8,25,26]. In detail, the peak at 2117 cm^−1^ is attributed to the C–O vibration on Pd^+^ [25]. Peaks in the region from 2130 cm^−1^ to 2155 cm^−1^ are assigned to the C–O vibration on Pd^2+^ ions bonded with the hydroxy of zeolites, and this Pd cationic species is named as naked Pd^2+^ by Gao et al. [8]. Note that an extra peak at 2179 cm^−1^ is observed in the spectrum of Pd/16 compared with the other two samples, which indicates the existence of another dispersed Pd cationic species in this sample. According to the studies of Gao [8] and Okumura [26] et al., this peak at 2179 cm^−1^ is attributed to the C–O vibration on Pd(OH)^+^. Since this species exists only in Pd/16 among these samples with the same Pd loading, the formation of Pd(OH)^+^ should be relative to the Si/Al ratio.

In addition, in situ FT-IR of CO adsorption was also carried out on Pd/16 treated by Na^+^ titration. As shown in Figure 5b, the disappearance of the peaks attributed to the C–O vibration on dispersed Pd cationic species is caused by the complete replacement of these Pd species by Na^+^. Meanwhile, the disappearance of CO-Pd^0^ is also observed. Considering the mild PH condition adopted, dispersed Pd^0^ should not be washed away if this species existed in samples before the titration process. So, dispersed Pd^0^ must be formed via CO reduction of dispersed Pd cationic species.

#### 3.3.5. XPS

**Figure 6:**
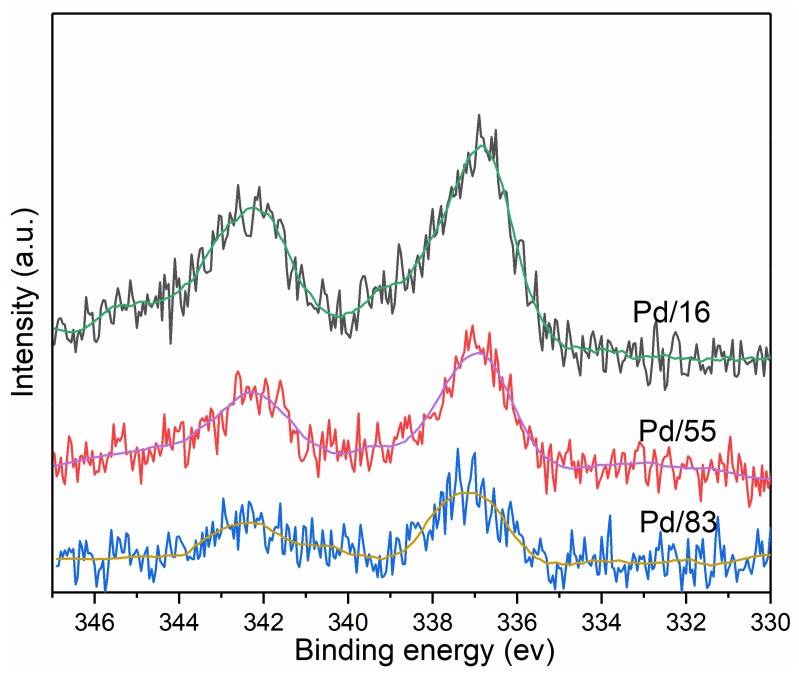
Pd3d X-ray photo electron spectroscopy (XPS) spectra of each sample.

Figure 6 shows the Pd3d XPS spectra of each sample. All samples display binding energy at 336.8 eV and 342.3 eV corresponding to PdO [8,27]. XPS results reveal that Pd^0^ and Pd^+1^ are absent in all samples, which can demonstrate the conclusion of in situ FT-IR of CO adsorption that Pd^0^ and Pd^+1^ were formed from CO reduction of dispersed Pd cationic species.

#### 3.3.6. In Situ FT-IR of NH_3_ Adsorption

**Figure 7:**
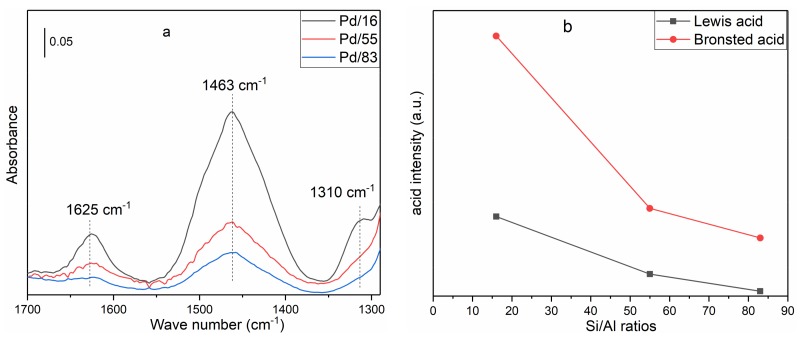
(**a**) In situ FT-IR of NH_3_ adsorption (temperature: 80 °C; flow: NH_3_ (500 ppm) + N_2_). (**b**) The acid intensity as a function of the Si/Al ratio.

Figure 7 shows the FT-IR spectra of ammonia adsorbed on each sample. The band at 1463 cm^−1^ is attributed to symmetric bending vibration of NH_4_^+^ bound to the Brønsted acid site and the bands at 1310 and 1625 cm^−1^ are attributed, respectively, to symmetric and antisymmetric deformation vibration of NH_3_ linked to the Lewis acid site [27,28,29,30]. As show in Figure 7a, the intensity of Brønsted acid and Lewis acid decreases with the increase of the Si/Al ratio. Besides, the sample of Pd/16 possesses two kinds of Lewis acid corresponding to the bands at 1310 and 1625 cm^−1^, and the samples of Pd/55 and Pd/83 only have the latter one. The peaks of Brønsted acid and Lewis acid were integrated, which can be used to compare Brønsted and Lewis acid intensity of the sample with different Si/Al ratios [31,32], and the result is presented in Figure 7b.

#### 3.3.7. Na^+^ Titration

The Pd species of Pd/Beta consist of Pd ions at exchange sites of zeolites (dispersed Pd ions, marked as Pd^δ+^) and aggregated palladium oxide (PdO_x_). Amounts of Pd ions at exchange sites of zeolites were quantified using Na^+^ titration as first reported by Kikuchi et al. [33]. Na^+^ can only exchange dispersed Pd ions and aggregated palladium oxide cannot be washed away. The amount of aggregated palladium oxide was quantified by ICP and the amount of dispersed Pd ions was calculated based on Equation (1).

(1) $$C_{{\mathrm{Pd}}^{\delta +}}\;=\;C_{\mathrm{total}}\;-\;C_{{\mathrm{PdO}}_{x}}$$

where $C_{{\mathrm{Pd}}^{\delta +}}$ and $C_{{\mathrm{PdO}}_{x}}$ are amounts of dispersed Pd ions and aggregated palladium oxide respectively, and $C_{\mathrm{total}}$ is the total amount of Pd loaded on samples. As shown in Table 4, with the increase of the Si/Al ratio, the amount of dispersed Pd ions decreases, whereas that of aggregated palladium oxide increases. 

## 4. Discussion

### 4.1. The Relationship of Pd Species and the NO Desorption Peak Centered at 130 °C of Pd/16

Comparing Figure 7 and Figure S4, we can see that the intensity of Brønsted acid decreases and two kinds of Lewis acid sites generate due to Pd^2+^ occupying exchange sites of zeolites, which indicates that the formation of two kinds of Lewis acid sites is related to Pd species. From the definition of Brønsted acid and Lewis acid, we know that the Pd^2+^ and Pd(OH)^+^ at exchange sites of zeolites are Lewis acid sites, and the Si–OH–Al of zeolites is a Brønsted acid site. As shown in Figure 5a, the peak at 2179 cm^−1^, attributed to the C–O vibration on Pd(OH)^+^, only exists in Pd/16 among these samples. Because the band at 1310 cm^−1^ in Figure 7 and Pd(OH)^+^ only exist in Pd/16 and Lewis acid is related to Pd species, the bands at 1310 and 1625 cm^−1^ might be attributed to NH_3_ adsorbed on Pd(OH)^+^ and Pd^2+^ at exchange sites of zeolites, respectively. 

Wang et al. [34] suggested that the bond interaction between the acidic proton of Brønsted acid and the oxygen atom of PdO led to the formation of Pd(OH)^+^. In addition, Okumura et al. [35] suggested the dispersion of PdO was determined by the acid base interaction between acid sites of zeolite and basic PdO. In other words, the formation of highly dispersed PdO depends on the intensity of acid sites in the zeolites. Therefore, it is reasonable to say that Pd(OH)^+^ only exists in Pd/16.

As shown in Figure 1b, two NO desorption peaks centered at 130 °C and 210 °C respectively are observed in the desorption stage of Pd/16, whereas NO is only desorbed at approximate 210 °C for Pd/55 and Pd/83. The operation temperature of downstream NH_3_-SCR catalysts is more than 200 °C. So these NO desorbed at 130 °C cannot be solved. Considering that Pd(OH)^+^ only exists in Pd/16, this additional NO desorption peak centered at 130 °C is likely to be caused by NO adsorption on Pd(OH)^+^. Okumura et al. [26] reported that the adsorption of NO onto the highly dispersed PdO caused the significant change in the local structure and the valence of Pd. Therefore, the structure of NO attached to Pd(OH)^+^ may be unstable comparing with NO attached to naked Pd^2+^, which may be the reason for NO desorption at low temperature on Pd/16. 

### 4.2. Conversion of NO and C_3_H_6_ in the Desorption Stage

From the discussion of Section 3.1, we can conclude that Pd/Beta can catalyze NO reduction and the C_3_H_6_ oxidation reaction in the desorption stage. Because the desorption temperature of NO and C_3_H_6_ are different, NO cannot react directly with C_3_H_6_. Since CO and N_2_O are released at the same temperature range and NO can be readily reduced by CO [36,37,38], NO is likely to be partially reduced to N_2_O by CO, and further reduced to N_2_, of which the formation is not capable of being measured under the conditions adopted.

**Figure 8:**
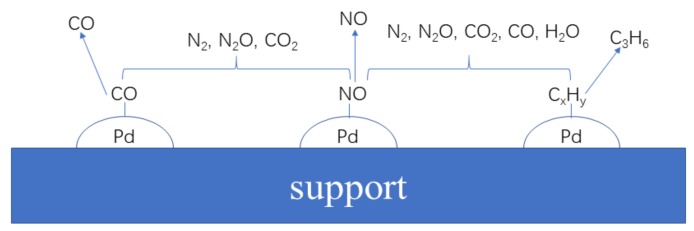
Scheme of the reaction paths in desorption stages for Pd/Beta.

According to Table 2, the desorption amounts of CO are more than the adsorption amounts of each sample, that is to say additional CO is formed in the desorption stage. In view of the strongest reducibility of CO among these gaseous components adopted, additional CO must be the product of the oxidation reaction. Since carbon deposition is widely observed in large-pore zeolites such as Beta [39], residual C_3_H_6_ which is not desorbed at approximately 110 °C should be deposited in samples. With the temperature increasing, these deposited carbon species are like to be partially oxidized to CO [40,41]. In this case, the formation of additional CO is explained to some extent. According to the above discussion, the scheme of reaction paths in desorption stages was drawn in Figure 8.

### 4.3. Influence of Si/Al Ratios on Cold-Start Catalyst Performance of Pd/Beta

According to in situ FT-IR of NH_3_ adsorption on each parent H-Beta (see Figure S4 in Supplementary Materials), the band at 1463cm^−1^ attributed to the vibration of NH_4_^+^ on Brønsted hydroxyl groups [28,29,30] becomes weaker when the Si/Al ratio increases, which indicates the lower content of cation exchange sites. Rice et al. [42] suggested that the Al–Al pair in zeolites is necessary for the formation of naked Pd^2+^, and the amount of the Al–Al pair is decreased exponentially at high Si/Al ratios. In this case, although the formation of Pd(OH)^+^ which can be balanced with one exchange site still needs to be further studied, the decrease of dispersed Pd ions in Pd/Beta with higher Si/Al ratios can be explained by a lower amount of paired exchange sites in the corresponding parent H-Beta. 

Because the intensity of Brønsted acid determines the amount of Pd ions at exchange sites that is the main Lewis acid of Pd/Beta, it can be explained that the Lewis acid intensity of Pd/Beta decreases with the increase of Si/Al ratios. As is well known, NO_x_ can only adsorb on Lewis acid sites in the presence of H_2_O [7,8,9,10], while C_3_H_6_ can adsorb on Brønsted acid and Lewis acid sites of Pd/Beta [43,44,45,46], although the majority of Brønsted acid sites are occupied by H_2_O. Therefore, with the increase of the Si/Al ratio, the decrease of Brønsted acid and Lewis acid intensity leads to the decrease of adsorption amounts of NO and C_3_H_6_.

**Figure 9:**
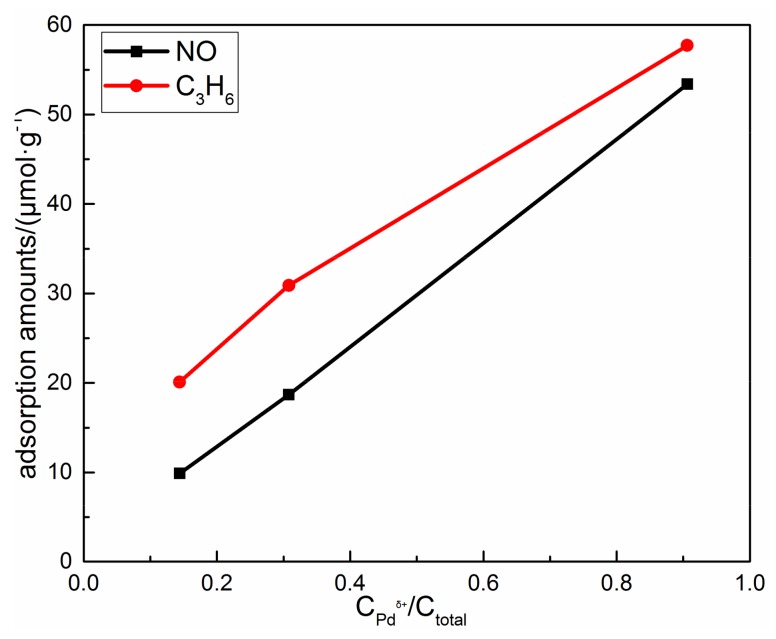
The relationship of NO and C_3_H_6_ adsorption amounts and the amount of dispersed Pd ions.

Figure 9 shows that NO and C_3_H_6_ adsorption amounts increase linearly with the increase of the amount of dispersed Pd ions, which indicates dispersed Pd ions might be the main active sites for NO and C_3_H_6_ adsorption in the adsorption stage. As reported by Chen [7] and Gao [8] et al., dispersed Pd cationic species are the main active sites for NO trapping in this catalyst. Meanwhile, Azambre et al. [43,44,45,46] reported that cations on exchange sites of zeolites are capable of trapping C_3_H_6_. So, this kind of Pd species is likely to be the active site for C_3_H_6_ trapping in Pd/Beta, too. Therefore, the decrease of NO and C_3_H_6_ adsorption amounts with higher Si/Al ratios can be explained by the lower amount of dispersed Pd ions. In addition, smaller Si/Al ratios also result in the formation of Pd(OH)^+^ from which adsorbed NO released at a much lower temperature, which can be solved by increasing Si/Al ratios of zeolite. In this respect, Pd/Beta with a Si/Al ratio = 55 is more appropriate for the cold-start application due to the balanced performance among the adsorption amounts and desorption temperature.

## 5. Conclusions

In this work, dispersed Pd ions quantified in Pd/Beta served as cold-start catalysts. Owing to the decrease of paired exchange sites in the corresponding parent H-Beta, the amount of dispersed Pd ions that were identified as main active sites for NO and C_3_H_6_ adsorption decreased in parallel with the increase of the Si/Al ratio. Moreover, the intensity of Brønsted and Lewis acid decreases with the increase of Si/Al ratios, which can also lead to the decrease of NO and C_3_H_6_ adsorption amounts. Therefore, Pd dispersion and acidic properties of Pd/Beta together determine the adsorption ability of NO and C_3_H_6_. In addition, the formation of an additional dispersed Pd cationic species, Pd(OH)^+^, led to earlier NO desorption in the low Si/Al ratios sample, which can be solved by increasing Si/Al ratios of zeolite. According to these studies above, an optimum Si/Al ratio of Pd/Beta was found at around 55 due to the balanced performance between the adsorption amounts and desorption temperature.

## Figures and Tables

**Table 1:** Loading and Si/Al ratios of each sample.

Sample	Si/Al Ratio	Pd Loading (wt.%)
Pd/16	16.20	0.96
Pd/55	54.52	1.04
Pd/83	82.81	0.97

**Table 2:** Adsorption amounts and desorption amounts of each gaseous component.

Sample	Adsorption Amount (μmol/gcat) ^a^			Desorption Amount (μmol/gcat)		
	NO	C_3_H_6_	CO	NO	C_3_H_6_	CO
Pd/16	53.4	57.7	28.6	20.1	23.2	33.1
Pd/55	18.7	30.9	7.9	6.6	10.7	29.9
Pd/83	9.9	21.1	5.4	1.5	5.7	9.1

^a^ The gcat indicates per gram catalyst.

**Table 3:** Textural properties of H-Beta and Pd/Beta samples.

Sample	S_BET_ (m^2^/g)	V_micro_ (cm^3^/g)
H-Beta/16	682.8	0.181
H-Beta/55	667.5	0.176
H-Beta/83	625.4	0.163
Pd/16	586.5	0.158
Pd/55	571.3	0.153
Pd/83	540.1	0.141

**Table 4:** Amounts of dispersed Pd ions and aggregated palladium oxide in each sample.

Sample	$C_{{\mathrm{Pd}}^{\delta +}}\;(\mathrm{wt}.\%)$	$C_{{\mathrm{PdO}}_{x}}(\mathrm{wt}.\%)$
Pd/16	0.87	0.09
Pd/55	0.32	0.72
Pd/83	0.14	0.83

## Supplementary Materials

The following are available online at https://www.mdpi.com/1996-1944/12/7/1045/s1, Figure S1: N2 adsorption-desorption isotherm of H-Beta (a) and Pd/Beta (b) samples with different Si/Al ratios. Figure S2: Pore size distribution curves of H-Beta (a) and Pd/Beta (b) samples with different Si/Al ratios. Figure S3: HRTEM figures of each sample (a: Pd/16; b: Pd/55; c: Pd/83). Figure S4: In situ FT-IR of NH3 adsorption (temperature: 80 °C; flow: 500 ppm NH_3_ + N_2_).
