# Supplementary material for: The Influence of Si/Al Ratios on Adsorption and Desorption Characterizations of Pd/Beta Served as Cold-Start Catalysts
Source: Materials (Basel). 2019 Mar 29;12(7):1045. doi: 10.3390/ma12071045 (PMC6479726; doi:10.3390/ma12071045)
Supplement: Supplementary file 1 [file materials-12-01045-s001.pdf]

materials

Supplementary Materials

The Influence of Si/Al Ratios on Adsorption and Desorption Characterizations of Pd/Beta Served as Cold-Start Catalysts

Ming Jiang [1], Jun Wang [1], Jianqiang Wang [1] and Meiqing Shen [1,2,3,*]

[1] Key Laboratory for Green Chemical Technology of State Education Ministry, School of Chemical Engineering& Technology, Tianjin University, Tianjin 300072, China; jiangming@tju.edu.cn (M.J.); wangjun@tju.edu.cn (J.W.); jianqiangwang@tju.edu.cn (J.W.)
[2] Collaborative Innovation Center of Chemical Science and Engineering (Tianjin), Tianjin 300072, China
[3] State Key Laboratory of Engines, Tianjin University, Tianjin 300072, China
* Correspondence: mqshen@tju.edu.cn

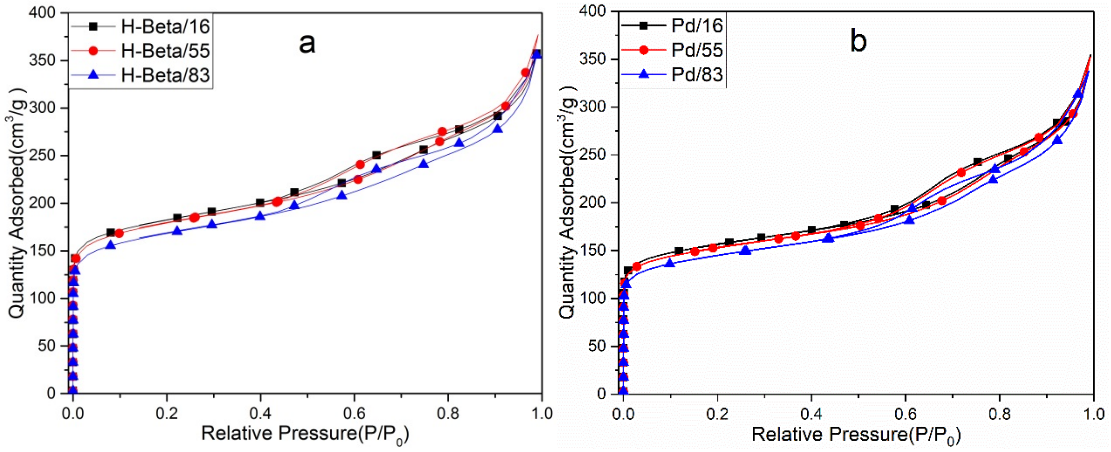

Figure S1. N_2 adsorption-desorption isotherm of H-Beta (a) and Pd/Beta (b) samples with different Si/Al ratios.

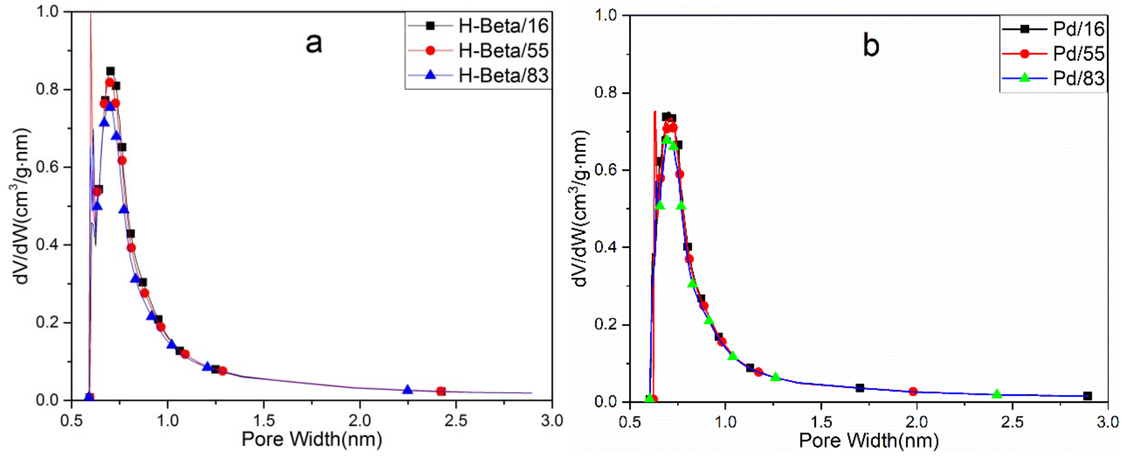

Figure S2. Pore size distribution curves of H-Beta (a) and Pd/Beta (b) samples with different Si/Al ratios.

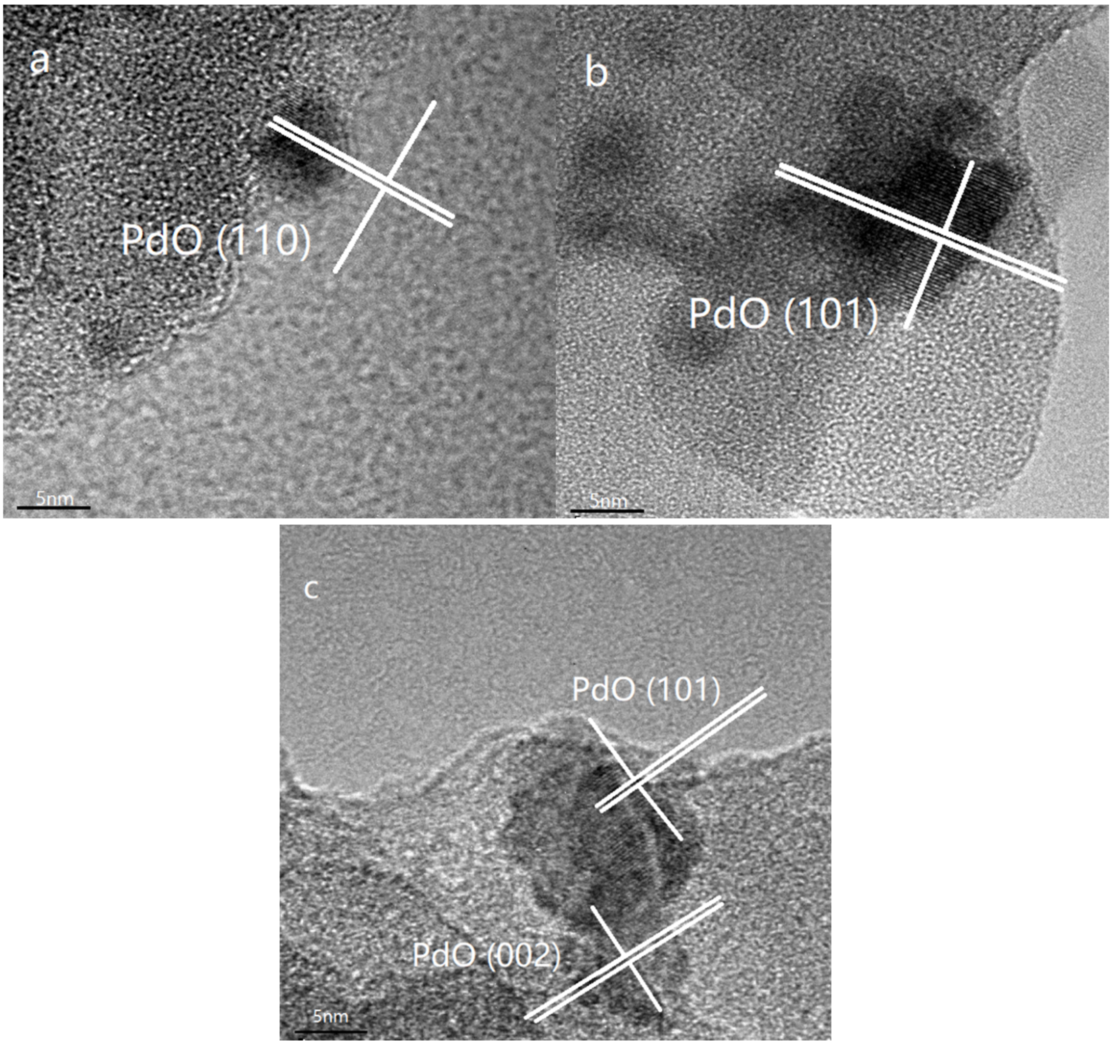

Figure S3. HRTEM figures of each sample (a: Pd/16; b: Pd/55; c: Pd/83).

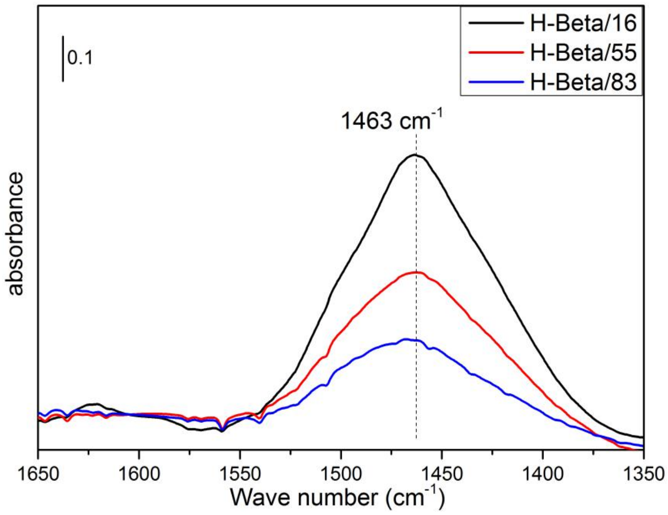

Figure S4. In situ FT-IR of NH_3 adsorption (Temperature: 80 °C; Flow: 500 ppm NH_3 + N_2).

© 2019 by the authors. Licensee MDPI, Basel, Switzerland. This article is an open access article distributed under the terms and conditions of the Creative Commons Attribution (CC BY) license (http://creativecommons.org/licenses/by/4.0/).
